# Autophagy-deficient breast cancer shows early tumor recurrence and escape from dormancy

**DOI:** 10.18632/oncotarget.25197

**Published:** 2018-04-24

**Authors:** Hussein F. Aqbi, Liliya Tyutyunyk-Massey, Rebecca C. Keim, Savannah E. Butler, Theresa Thekkudan, Supriya Joshi, Timothy M. Smith, Dipankar Bandyopadhyay, Michael O. Idowu, Harry D. Bear, Kyle K. Payne, David A. Gewirtz, Masoud H. Manjili

**Affiliations:** ^1^ Department of Microbiology and Immunology, Virginia Commonwealth University School of Medicine, Richmond, 23298, VA, USA; ^2^ Department of Pharmacology and Toxicology, Virginia Commonwealth University School of Medicine, Richmond, 23298, VA, USA; ^3^ Massey Cancer Center, Virginia Commonwealth University School of Medicine, Richmond, 23298, VA, USA; ^4^ Department of Human and Molecular Genetics, Virginia Commonwealth University School of Medicine, Richmond, 23298, VA, USA; ^5^ Department of Biostatistics, Virginia Commonwealth University School of Medicine, Richmond, 23298, VA, USA; ^6^ Department of Pathology, Virginia Commonwealth University School of Medicine, Richmond, 23298, VA, USA; ^7^ Department of Surgery, Virginia Commonwealth University School of Medicine, Richmond, 23298, VA, USA; ^8^ Department of Immunology, Moffitt Cancer Center, Tampa, 33612, FL, USA; ^9^ VCU Institute of Molecular Medicine, Virginia Commonwealth University School of Medicine, Richmond, 23298, VA, USA

**Keywords:** breast cancer, autophagy, tumor dormancy, tumor escape and relapse, cancer immunotherapy

## Abstract

Breast cancer patients who initially respond to cancer therapies often succumb to distant recurrence of the disease. It is not clear why people with the same type of breast cancer respond to treatments differently; some escape from dormancy and relapse earlier than others. In addition, some tumor clones respond to immunotherapy while others do not. We investigated how autophagy plays a role in accelerating or delaying recurrence of neu-overexpressing mouse mammary carcinoma (MMC) following adriamycin (ADR) treatment, and in affecting response to immunotherapy. We explored two strategies: 1) transient blockade of autophagy with chloroquine (CQ), which blocks fusion of autophagosomes and lysosomes during ADR treatment, and 2) permanent inhibition of autophagy by a stable knockdown of ATG5 (ATG5^KD^), which inhibits the formation of autophagosomes in MMC during and after ADR treatment. We found that while CQ prolonged tumor dormancy, but that stable knockdown of autophagy resulted in early escape from dormancy and recurrence. Interestingly, ATG5^KD^ MMC contained an increased frequency of ADR-induced polyploid-like cells and rendered MMC resistant to immunotherapy. On the other hand, a transient blockade of autophagy did not affect the sensitivity of MMC to immunotherapy. Our observations suggest that while chemotherapy-induced autophagy may facilitate tumor relapse, cell-intrinsic autophagy delays tumor relapse, in part, by inhibiting the formation of polyploid-like tumor dormancy.

## INTRODUCTION

Autophagy plays a paradoxical role in the promotion and inhibition of cancer. On the one hand, autophagy has a cancer-promoting role by protecting tumor cells from chemotherapy or providing a source of energy for tumor cells to survive under hypoxic and acidic conditions despite the lack of mature vessels [1]. On the other hand, inhibition of autophagy by disruption of *Beclin 1* or deletion of *ATG5* increases the frequency of spontaneous malignancies [2] or liver tumor [3], respectively. Recently, four different mechanisms have been proposed to describe paradoxical functions of autophagy in cancer, which include cytotoxic, cytostatic, cytoprotective and non-protective autophagy [4]. There are also three major types of autophagy which include micro-autophagy involving the direct engulfment of cytosolic material by lysosomes through invagination, chaperone-mediated autophagy involving HSP70 and the lysosomal membrane associated protein 2 A (LAMP2A), and macro-autophagy which is a highly conserved pathway involving the formation of autophagosomes, which fuse with lysosomes. To this end, ATG5 is involved in the elongation of autophagosomes to engulf toxic material for degradation. A stable knockdown of ATG5 results in the inhibition of the formation of autophagosomes and progression of macro-autophagy [5]. Chloroquine (CQ), on the other hand, does not have any effects on autophagosomes but it blocks the fusion of autophagosomes and lysosomes, thereby preventing the completion of macro-autophagy. In order to investigate the role of macro-autophagy in tumor dormancy and relapse, we performed a transient inhibition of macro-autophagy by means of CQ during chemotherapy, which mainly inhibits chemotherapy-induced autophagy while cell-intrinsic autophagy will be restored after the completion of chemotherapy. We also performed a permanent inhibition of cell-intrinsic macro-autophagy by a stable knockdown of ATG5 in tumor cells. We demonstrated that cell-intrinsic, but not chemotherapy-induced, autophagy can inhibit tumor relapse.

## RESULTS

### Adriamycin induces autophagy in MMC

**Figure 1:**
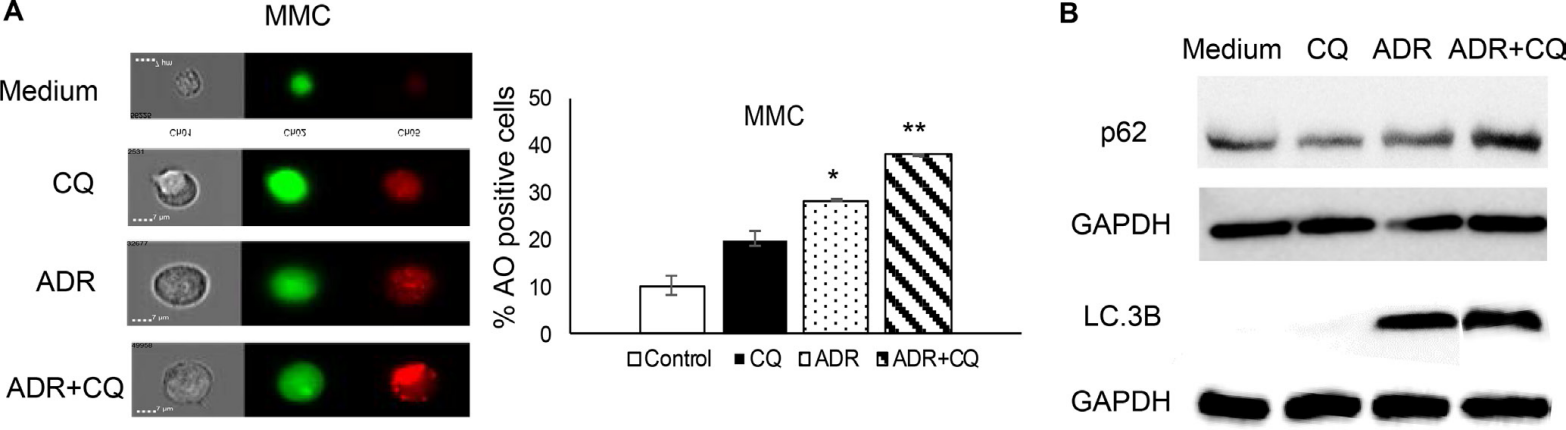
CQ blocks ADR-induced autophagy MMC tumor cells received three daily doses of ADR alone (1 µM ADR for 2 hrs) (ADR) or in the presence of CQ (10 µM 3 hrs before ADR and 2 hrs during ADR treatment) (ADR+CQ), washed after each daily treatment and analyzed by acridine orange (AO) one day after the last treatment. Untreated MMC (Medium) or MMC treated with CQ (CQ) served as controls. (**A**) Acridine orange (AO) staining was analyzed for acidic vesicles (red) using image cytometry. Data represent triplicate experiments. (**B**) Levels of p62/SQSTM1 and LC.3B after treatment with ADR ± CQ indicative of autophagy induction in the absence of autophagic flux (B).

In order to determine whether ADR induces autophagy and in turn establishes tumor dormancy, MMC cells were treated with ADR in the presence or absence of CQ, a pharmacological agent used to block the final stages of autophagy, specifically the fusion of autophagosomes with lysosomes that is necessary for digestion of the cargo in the autophagosomes (frequently termed “autophagic flux”). CQ blocked this autophagic flux as evidenced by the enhanced accumulation of acidic vesicles (red signals) (Figure 1A, ADR and ADR+CQ). We further monitored degradation of the p62/SQSTM1 protein as a marker of autophagic flux, and LC.3B expression as a marker of autophagosomes formation (since LC3 is a component of the autophagosomes). As shown in Figure 1B, ADR did not induce degradation of p62/SQSTM1 although it elevated LC.3B, suggesting that ADR induces autophagy but fails to drive autophagy to completion and p62/SQSTM1 degradation.

### A transient blockade of autophagy by CQ during ADR treatment delays tumor relapse *in vitro* but not *in vivo*

**Figure 2:**
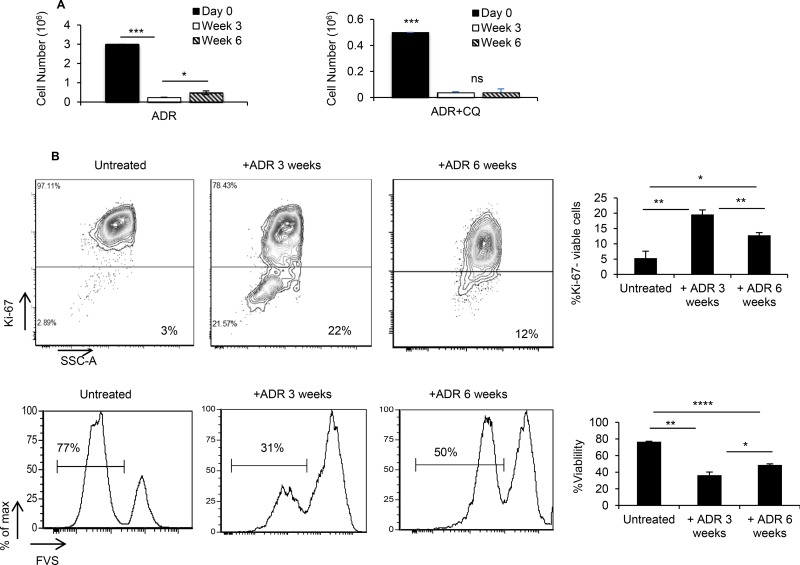
ADR-induced dormant tumor cells remain dormant in the presence of CQ MMC tumor cells were treated with 3 daily doses of ADR (1 uM for 2 hrs), with one group receiving CQ (10 uM) 3 hrs prior to and during ADR treatment. Both groups remained untreated for 3 weeks and 6 weeks, *in vitro*. (**A**) Adherent viable cells were counted using trypan blue exclusion at various time points. Data represent 3 replicates ± SEM. (**B**) At weeks 3 and 6 post-treatment, Ki-67 expression (upper panel) and viability (lower panel) were quantified within the population of adherent tumor cells. Data represent 2–3 replicates ± SEM. Four independent experiments have been carried out which have shown similar results.

Since CQ is being used to sensitize tumor cells susceptible to chemotherapy [6], we sought to determine whether blockade of autophagy by CQ during ADR treatment affects tumor dormancy and relapse. We showed that the presence of CQ during ADR treatment, *in vitro*, resulted in prolonging tumor dormancy such that, while ADR treated MMC resumed cell proliferation 6 weeks after the treatment, ADR+CQ treated MMC remained dormant (Figure 2A). In order to confirm tumor cell relapse after 6 weeks, flow cytometry analysis of ADR-treated MMC was performed, and indicated a shift of Ki67- non-proliferating cells to Ki67+ proliferating cells with a greater viability (Figure 2B). In fact, MMC cells remained apoptotic by producing floater dead cells following ADR treatment (Supplementary Figure 1A) which compensated for cell proliferation and maintained tumor dormancy for 3 weeks after the completion of ADR treatment. Follow up studies on floater cells showed they were all apoptotic (Supplementary Figure 1B). A transient blockade of autophagy by CQ did not affect susceptibility of tumor cells to ADR-induced apoptosis (Supplementary Figure 2). On the other hand, a transient blockade of autophagy during ADR chemotherapy, *in vivo*, did not prolong tumor dormancy in FVBN202 mice (Supplementary Figure 3).

### A transient blockade of autophagy by CQ during ADR treatment does not change susceptibility of tumor cell to immunotherapy

**Figure 3:**
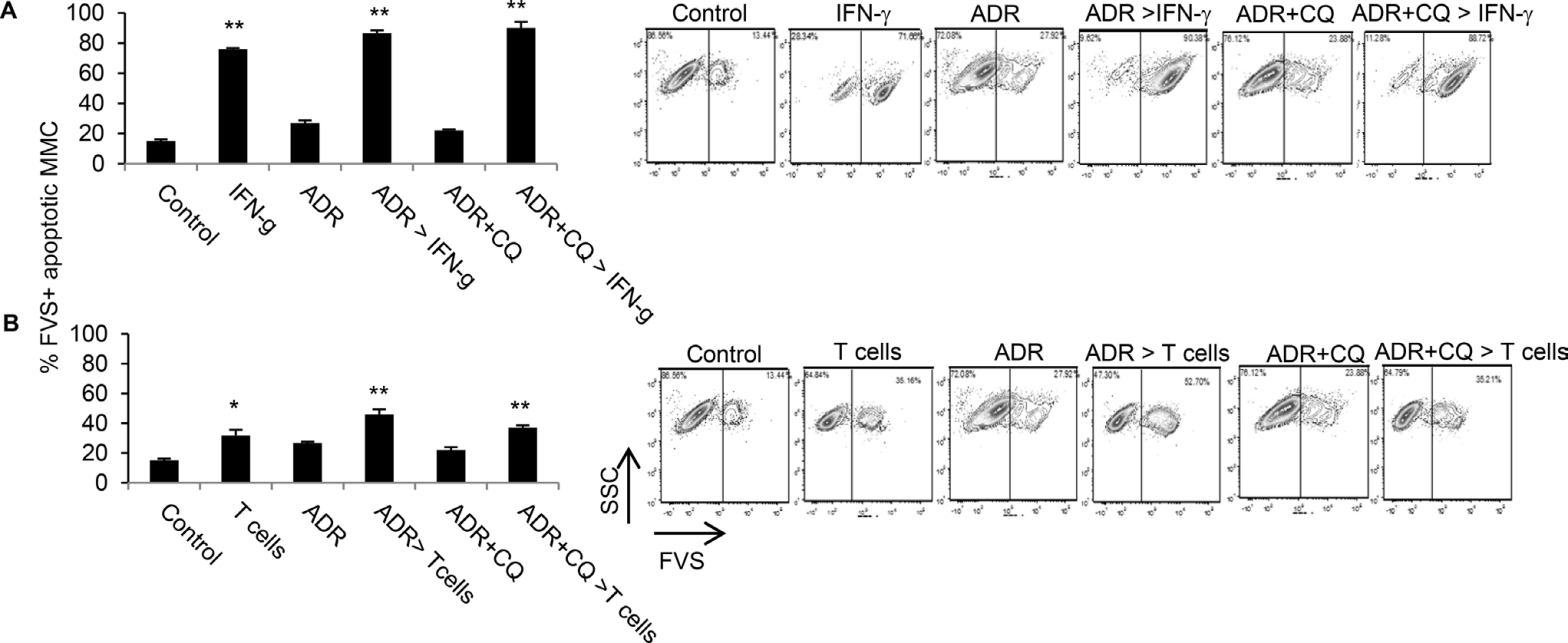
Dormant tumor cells established by ADR or ADR+CQ remain susceptible to immunotherapy The *in vitro* tumor dormancy was established three weeks after three daily treatments of MMC with ADR or ADR+CQ. Untreated MMC cells were used as control. (**A**) Apoptosis was determined by FVS viability staining in MMC (control), ADR-treated dormant MMC (ADR), ADR+CQ-treated dormant MMC (ADR+CQ), as well as control MMC cultured with three daily doses of IFN-g and analyzed two days later (50 ng/ml) (IFN-g), ADR-treated dormant MMC cultured with three daily doses of IFN-g (50 ng/ml) and analyzed two days later (ADR > IFN-g), or ADR+CQ-treated dormant MMC cultured with three daily doses of IFN-g (50 ng/ml) and analyzed two days later (ADR+CQ > IFN-g). (**B**) Apoptosis was determined by FVS viability staining of MMC (control), MMC cultured with MMC-sensitized T cells for 48 hrs (T cells), ADR-treated dormant MMC (ADR), ADR-treated dormant MMC cultured with MMC-sensitized T cells for 48 hrs (ADR > T cells), ADR+CQ-treated dormant MMC (ADR+CQ), or ADR+CQ-treated dormant MMC cultured with MMC-sensitized T cells for 48 hrs (ADR+CQ > T cells). Splenic T cells were collected from MMC tumor-bearing FVBN202 mice.

In order to determine whether a transient blockade of autophagy during ADR treatment affects susceptibility of dormant MMC to immunotherapy, dormant MMC were cultured with either IFN-γ or MMC-reactive T cells three weeks after treatment with ADR or ADR+CQ. As shown in Figure 3, untreated MMC or dormant MMC treated with ADR or ADR+CQ all remained susceptible to IFN-γ treatment or T cells.

### A stable knockdown of autophagy reduces susceptibility of MMC to ADR treatment

**Figure 4:**
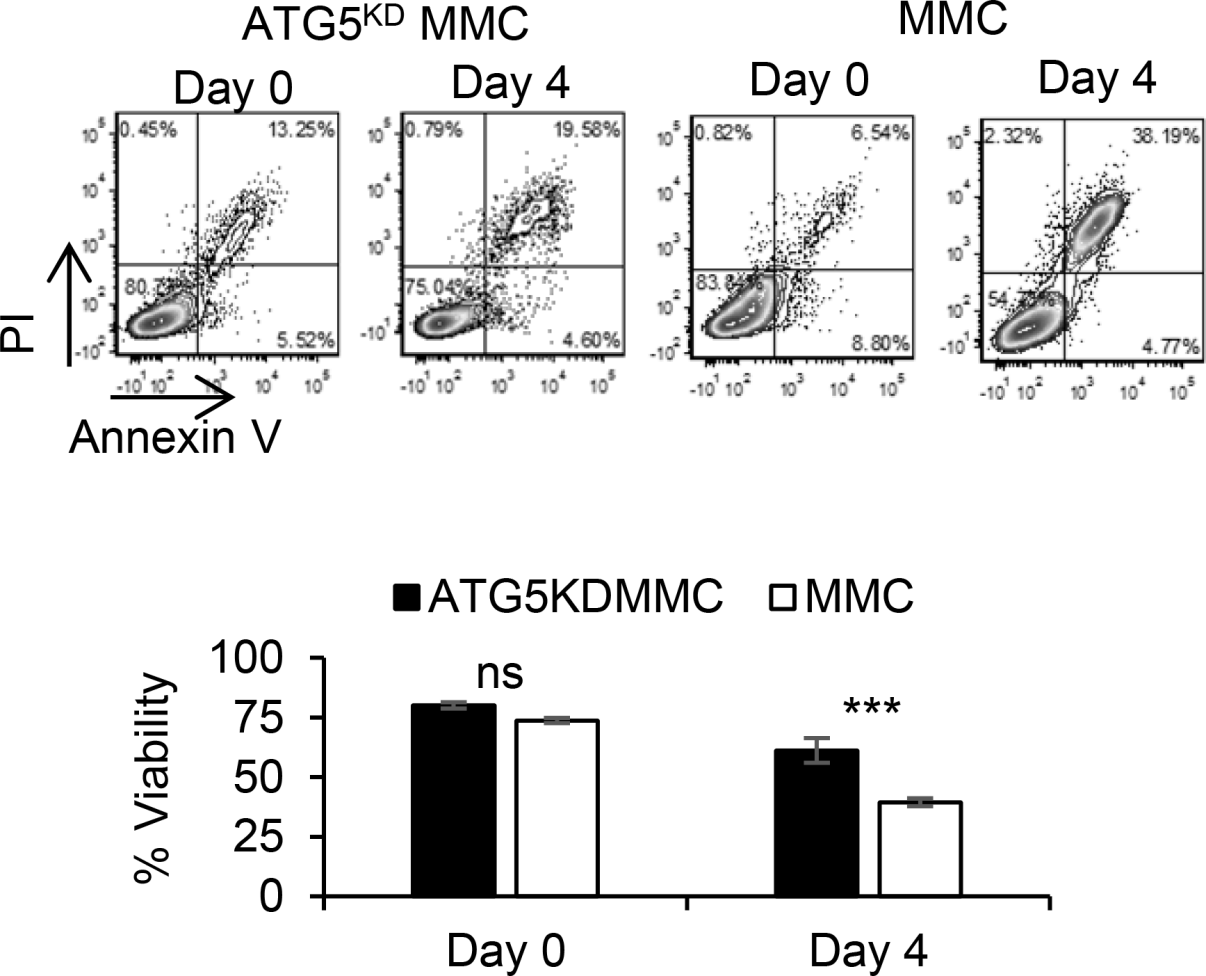
Autophagy knockdown tumor cells become less susceptible to ADR-induced apoptosis Autophagy-deficient MMC (ATG5^KD^ MMC) or autophagy-competent MMC (MMC) were treated with a single dose of ADR alone (1 uM ADR for 2 hrs). Tumor cells were analyzed by Annexin V/PI staining prior to treatment (Day 0) or three days after the treatment (Day 4). Experiments were performed in triplicates.

CQ only transiently blocks fusion of autophagosomes and lysosomes during ADR treatment such that after removal of CQ, accumulated autophagosomes could eventually be fused with lysosomes to complete autophagy. In order to determine the role of autophagy in tumor dormancy or relapse, we used shRNA for a stable knockdown of ATG5 (ATG5^KD^) which inhibits formation of autophagosomes in MMC. Scrambled shRNA was used as control (Supplementary Figure 4A). The ATG5^KD^ MMC and scrambled control MMC were irradiated to confirm that ATG5^KD^ MMC cells were deficient in autophagy, using p62 and LC.3B as read outs (Supplementary Figure 4B). Tumor cells remained intact for the expression of neu antigen, as well as cell proliferation *in vitro* and *in vivo* following knockdown of autophagy (Supplementary Figure 4C–4E). Flow cytometry analysis determined a lower level of viability in MMC compared with ATG5^KD^ MMC following ADR treatment (Figure 4).

### A stable knockdown of autophagy results in earlier tumor relapse associated with increased frequency of polyploid-like cells and resistance to immunotherapy

**Figure 5:**
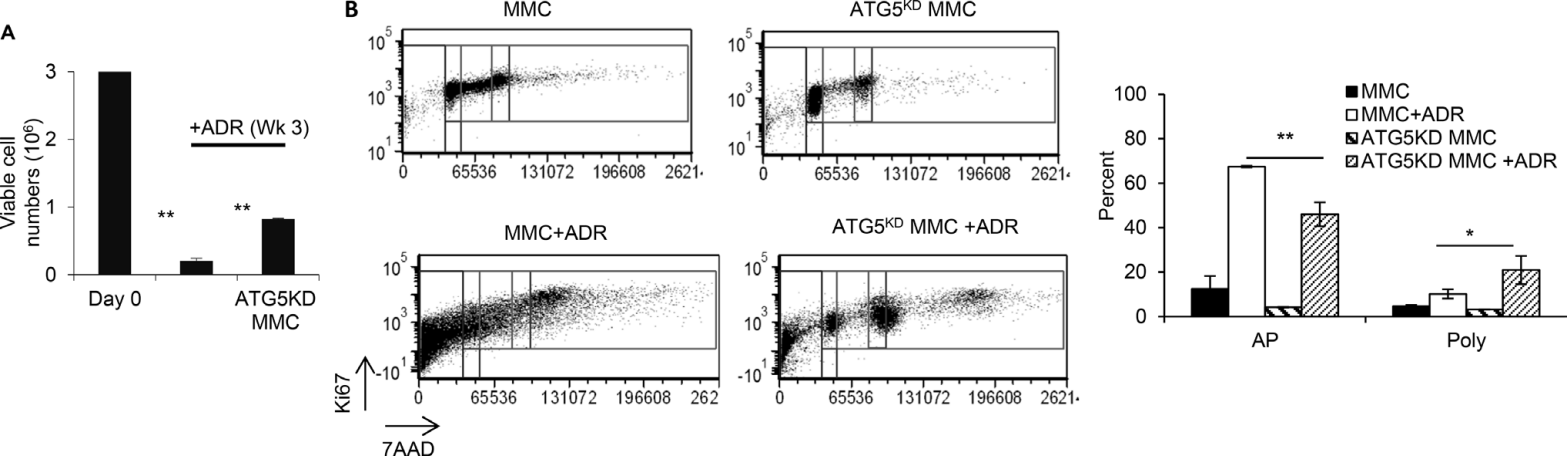
ADR-induced tumor dormancy in autophagy knockdown tumor cells with polyploid-like morphology compared with autophagy competent tumor cells, *in vitro* MMC or ATG5^KD^ MMC tumor cells (3 million cells, Day 0) were treated with 3 daily doses of ADR (1uM for 2 hrs), and viable cells were counted at week 3 using trypan blue exclusion. Data represent triplicate experiments (**A**). Dot plots from each experimental group gated for cell cycle phase based upon DNA content (7-AAD) and Ki-67 expression. Events falling to the left of the G1/G0 gates are considered apoptotic cells (AP). Events falling to the far right of the G2/M gate are considered polyploid-like cells (Poly) (**B**). Three independent experiments have been performed and data represent 3 replicates ± SEM.

In order to determine whether a higher viability of ATG5^KD^ MMC following ADR treatment (Figure 4) facilitates an earlier tumor relapse compared with wild type MMC, follow up studies were performed for three weeks after ADR treatment. As shown in Figure 5A, ATG5^KD^ MMC survived better than autophagy-competent MMC following ADR treatment showing a significantly higher number of cells by 3 weeks after the treatment. Flow cytometry analysis of tumor cells showed greater levels of apoptosis in wild type MMC compared with ATG5^KD^ MMC (Figure 5B, *p <* 0.001). Interestingly, ATG5^KD^ MMC cells contained a higher number of polyploid-like cells following ADR treatment compared with autophagy-competent MMC (Figure 5B, *p <* 0.03).

**Figure 6:**
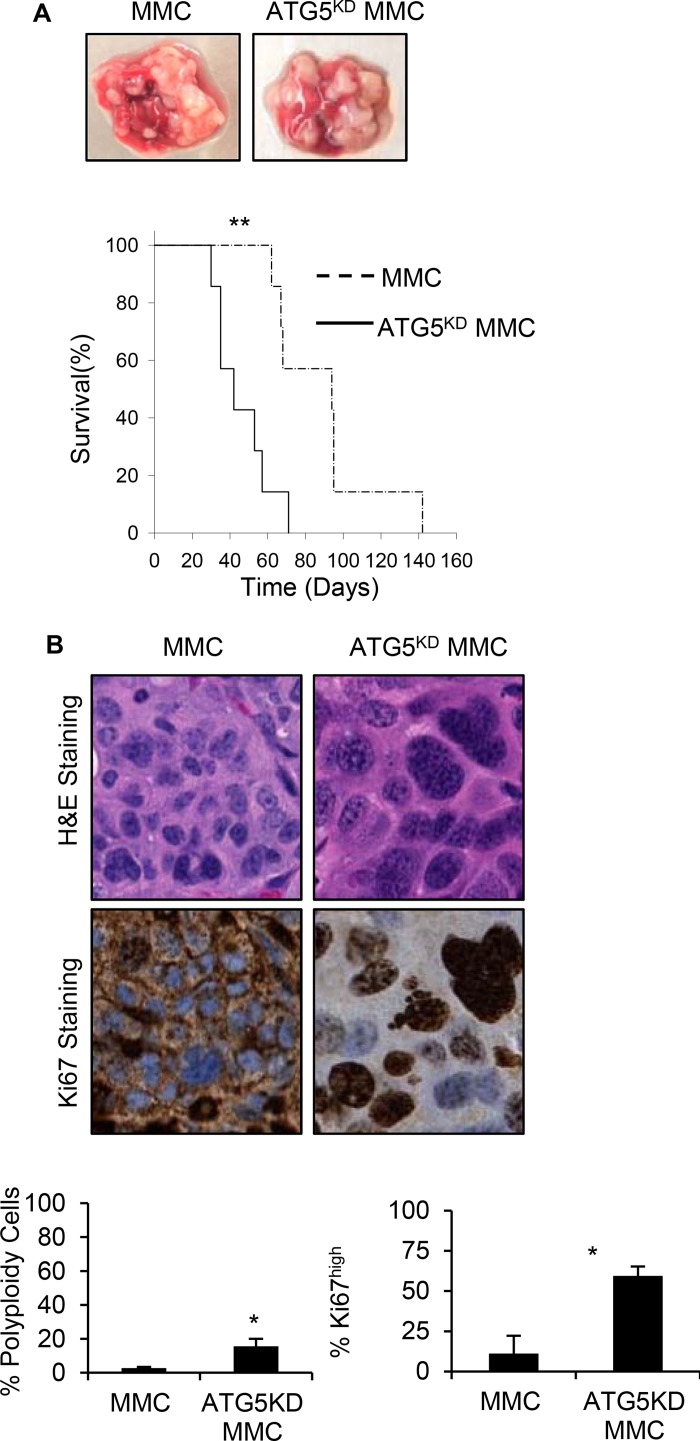
Earlier relapse of autophagy knockdown tumor cells with polyploid morphology compared with autophagy competent tumor cells, *in vivo* (**A**) FVBN202 mice (*n* = 7) were challenged i.v. with 10^6^ cells ADR-treated dormant control MMC (MMC), or ADR-treated dormant ATG5^KD^ MMC (ATG5^KD^ MMC). Animals were euthanized as soon as they became moribund. Representative tumor relapse in the lung and survival curve are shown. (**B**) Relapsed tumors were collected and immunohistochemistry slides were prepared by either staining samples with hematoxylin and eosin (H&E) or by Ki67 staining followed by subsequent digitization and analysis with NDP View software (Hamamatsu Photonics). At twenty-times magnification, three representative 0.02 mm^2^ areas were chosen from the H&E slides containing approximately 100 cells to measure nuclear envelope size. Cells containing a nuclear envelope equal to or greater than 16 um with visible multi-nuclei were considered polyploid-like or high grade cells. The corresponding cell was then analyzed on the Ki67 stained slide to determine Ki67 expression levels. Data was collected from three biological samples. Significance is based on a two-tailed *t*-test of *p* < 0.05.

**Figure 7:**
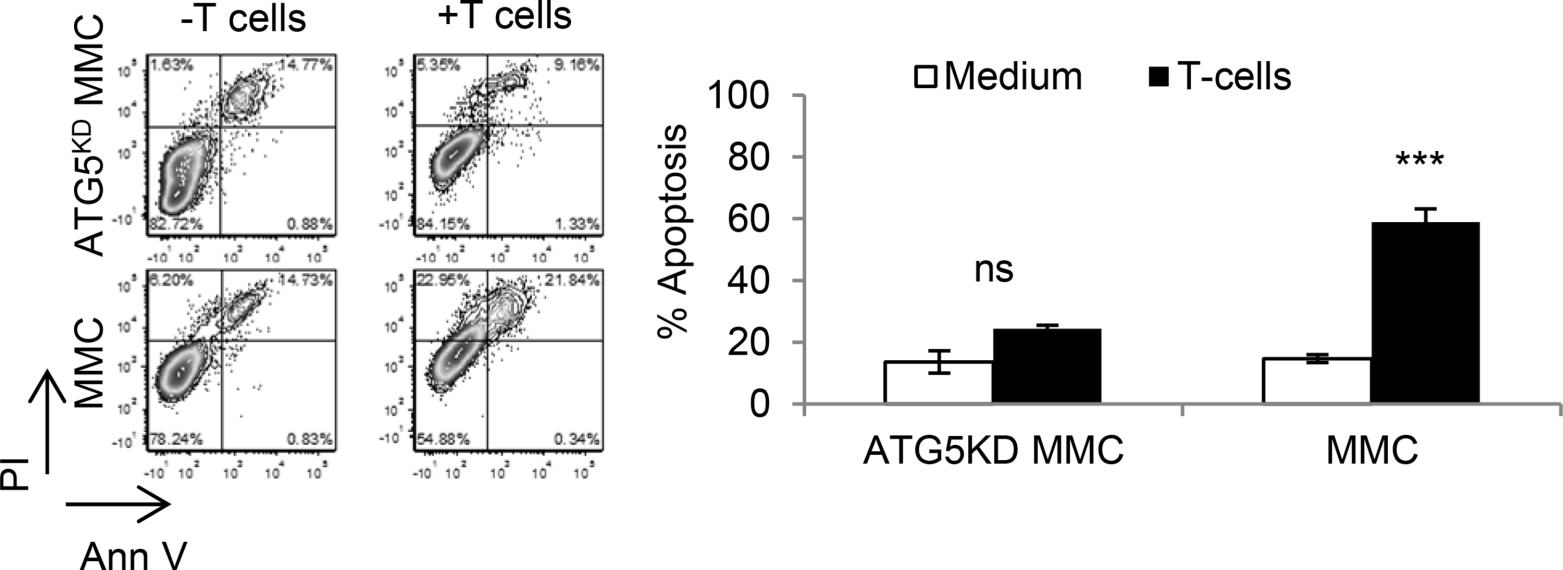
Autophagy knockdown tumor cells become resistant to T cell-induced apoptosis Neu overexpressing autophagy-deficient MMC (ATG5^KD^ MMC) or autophagy-competent MMC (MMC) were co-cultured with MMC-sensitized T cells and then gated CD45-Neu+ tumor cells were analyzed by Annexin V/PI staining. Data represents triplicate experiments.

In order to determine the *in vivo* relevance of our *in vitro* findings, FVBN202 mice were used. Tumor dormancy was first established by ADR treatment *in vitro*; FVBN202 mice (*n =* 7/group) were then challenged i.v. with one million viable dormant tumor cells. Animals were then sacrificed when they became moribund (lost 10% weight) as a result of massive lung metastasis. As can be seen in Figure 6A, animals that were challenged with ADR-treated ATG5^KD^ MMC developed lung metastasis significantly sooner than those that were challenged with ADR-treated MMC. Hematoxylin/eosin and immunohistochemistry analyses of tumor lesions determined a higher frequency of polyploid-like and Ki67+ tumor cells in animals that were challenged with ADR-treated ATG5^KD^ MMC (Figure 6B). Finally, ATG5^KD^ MMC were found to be resistant to T cell-induced apoptosis compared with autophagy-competent MMC (Figure 7).

## DISCUSSION

Cell-intrinsic autophagy is an ongoing process, which regulates cellular metabolism and homeostasis. Autophagy is also induced by insults such as chemotherapy. Here, we studied a paradoxical role of autophagy in tumor promotion and tumor inhibition by a transient inhibition of autophagy only during chemotherapy or a stable knockdown of autophagy in MMC tumor cells. While the former transiently blocked autophagy and cell-intrinsic autophagy was restored after the completion of chemotherapy, the latter permanently blocked chemotherapy-induced autophagy and cell-intrinsic autophagy. We demonstrated that inhibition of chemotherapy-induced autophagy by CQ did not increase susceptibility of tumor cells to chemotherapy-induced apoptosis. Nevertheless, chemotherapy-induced autophagy appeared to accelerate tumor relapse such that use of CQ during chemotherapy delayed tumor relapse *in vitro*. Our observation is consistent with other reports showing that increased autophagy in residual breast cancer after neoadjuvant chemotherapy was correlated with increased risk of tumor relapse [7]. A transient blockade of autophagy during chemotherapy of tumor-bearing animals did not affect tumor relapse, perhaps, because tumor inhibitory effects of *in vivo* chemotherapy was not as effective as *in vitro* drug treatment. Also, chemotherapy-induced autophagy did not affect the sensitivity of tumor cells to apoptosis induced by IFN-γ or tumor-reactive T cells.

We also demonstrated that, unlike chemotherapy-induced autophagy, cell-intrinsic autophagy accelerated tumor relapse. A stable knockdown of cell-intrinsic autophagy by ATG5 shRNA resulted in a reduced sensitivity of tumor cells to chemotherapy- or T cell-induced apoptosis, and accelerated tumor relapse *in vivo*. These effects coincided with an increased frequency of multinuclear polyploid-like dormant cells. These observations suggest that chemotherapy-induced autophagy could have tumor-promoting effects and facilitate tumor relapse, whereas cell-intrinsic autophagy could synergize with cancer therapeutics and delay tumor relapse. In fact, cell-intrinsic autophagy would seem to inhibit the formation of multinuclear cells following chemotherapy, and to prevent chemotherapy-induced genetic instability associated with resistance to cancer therapeutics. Similar observations have been made in other breast tumor models by showing that CQ but not knockdown of Beclin 1 or ATG12 sensitized the tumor to chemotherapy [8]. Therefore, anti-tumor effects of autophagy inhibitors such as CQ is likely to be because of the inhibition of chemotherapy-induced autophagy while anti-tumor effects of autophagy inducers such as rapamycin may result from enhanced cell-intrinsic autophagy [9, 10]. It has been reported cancer stem cells play a role in tumor dormancy [11] and drug resistance [12], and that immunotherapeutic targeting of breast cancer stem cells inhibits growth of mammary carcinoma [13]. However, we did not detect the enrichment of CD44+CD24- cancer stem cells following ADR-induced tumor dormancy (data not shown).

Anticancer drugs and ionizing radiation tend to induce autophagy in tumor cells [14]. Treatment-induced autophagy could lead to apoptosis [15] and tumor cell dormancy [16]. We have already reported that dormant tumor cells established by ADR treatment or radiation therapy, *in vitro*, developed resistance to these treatments but remained susceptible to immunotherapy [17]. Therefore, evaluation of apoptosis or tumor growth inhibition as a single factor without evaluating tumor dormancy and relapse may not be sufficient for understanding anti-cancer efficacy of autophagy inhibitors such as CQ. Inhibition of autophagy by CQ during chemotherapy diminishes the expression of DNA repair proteins, resulting in tumor growth inhibition in carboplatin-resistant BRCA1 wild-type TNBC orthotopic xenografts [18]. In triple negative breast cancer, CQ sensitizes tumor cells to paclitaxel chemotherapy [19]. In several tumor models, CQ synergistically augmented sunitinib cytotoxicity on tumor cells [6]. However, the role of CQ in inhibiting tumor recurrence has yet to be determined.

Cells that are deficient in autophagy show increased levels of reactive oxygen species which result in the accumulation of DNA damage, increased double-strand breaks and polyploid nuclei [20, 21]. To this end, cell-intrinsic autophagy protects the cell from genomic instability induced by the accumulation of toxins within the cell [22]. It has been reported that Beclin1 knockout mice fail to maintain genomic integrity by increasing DNA double stranded breaks and gene amplifications [20]. A higher expression of Beclin 1 in healthy breast tissue than in breast cancer suggests a deficiency in cell-intrinsic autophagy in tumors [23], which could contribute to genomic instability during tumorigenesis. In breast cancer patients who received adjuvant chemotherapy, presence of tumor cell intrinsic autophagy contributed to reduced risk of tumor relapse [24]. Expression of ATG5 in the tumor specimens is also associated with relapse-free survival in breast cancer patients [25]. In glioma, reduced tumor cell progression and relapse by knockdown of CDGSH iron sulfur domain 2 (CISD2) was associated with the activation of Beclin 1-mediated autophagy [26].

Our observations suggest that any deficiency in tumor cell-intrinsic autophagy could result in a reduced sensitivity of breast cancer to chemotherapy or immunotherapy. Therefore, IHC analysis of tumor biopsies before and after neoadjuvant or adjuvant chemotherapy could determine cell-intrinsic and chemotherapy-induced autophagy, respectively, and in turn might predict the risk of distant recurrence of the diseases accordingly. In future studies, other murine and human breast tumor cell lines as well as other types of carcinoma cells should be used in order to determine whether our findings offer a general mechanism of autophagy-associated tumor dormancy and relapse, or it might be a cancer specific phenomenon.

## MATERIALS AND METHODS

### Tumor cell line

The neu overexpressing mouse mammary carcinoma (MMC) cell line was established from spontaneous mammary tumors harvested from FVBN202 mice [27]. Tumor cells were maintained in RPMI 1640 supplemented with 10% FBS.

### Genetic silencing of ATG5 in MMC

Mission shRNA bacterial stocks for ATG5 and scrambled Control were purchased from Sigma Aldrich. Lentiviruses were produced in HEK 293TN cells co-transfected using Endo F ectinTM Lenti Transfection Reagent (GeneCopoeia, 1001–01) with a packaging mixture of psPAX2 and pMD2.G constructs (Addgene). Media containing the viruses was used to infect MMC cells; puromycin (1 μg/ml) was used as a selection marker to enrich for infected cells.

### Antibodies

All antibodies were purchase from Biolegend (San Diego, CA, USA) unless otherwise stated. Antibodies were used as instructed by the supplier. Antibodies include: anti-CD16/32 (clone 93), APC-anti-mouse IgG (Poly4053), PE-Ki67 (16A8), Alexa flour 488-Ki67 (11F6), Brilliant Violet 605-CD45 (30-F11), FITC-Annexin V, APC-Annexin V, 7-AAD viability staining solution and Propidium Iodide solution (PI), mouse anti-rat neu (anti–c-Erb2/c-Neu; 7.16.4, Calbiochem, Billerica, MA, USA), FITC-FVS (BD Biosciences). All reagents were used at the manufacturer’s recommended concentration.

### Mice

FVBN202 transgenic female mice (The Jackson Laboratory; Bar Harbor, ME, USA) were used. These mice overexpress non-mutated, non-activated rat neu transgene under the regulation of the mouse mammary tumor virus promoter [28]. These mice develop premalignant mammary hyperplasia similar to ductal carcinoma *in situ* prior to the development of spontaneous carcinoma [29]. These studies have been reviewed and approved by the Institutional Animal Care and Use Committee at Virginia Commonwealth University.

### Experimental tumor dormancy

*In vitro* tumor dormancy was established by the treatment of MMC or ATG5^KD^ MMC tumor cells with 3 daily doses of ADR (Sigma-Aldrich, 1uM for 2 hrs). During ADR treatment, MMC tumor cells were cultured without or with CQ (Sigma-Aldrich, 10 uM, 3 hrs prior to and during ADR treatment). By 2 weeks after the treatment, all groups did not show any increases in the number of adherent cells, which is the characteristic of tumor dormancy. For *in vivo* induction of tumor dormancy, FVBN202 mice were challenged with ADR-treated dormant MMC or ATG5^KD^ MMC (i.v. injection of 1 million viable cells), or untreated MMC followed by 3 weekly treatments of ADR (i.v., 9 mg/kg) or with 3 weekly treatment of ADR + 60 mg/kg CQ (i.p.).

### Cytotoxicity assay

Freshly isolated tumor-primed splenic T cells or *ex vivo* expanded splenic T cells were cultured with MMC at a 10:1 E:T ratio in 3 ml complete medium (RPMI-1640 supplemented with 100 U/ml of penicillin, 100 µg/ml streptomycin, 10% FBS, 10 mM L-glutamine and 5 × 10^–5^ M 2-mercaptoethanol) with 20U/ml of IL-2 (Peprotech) in 6 well culture dishes. After 48 hs cells were harvested and stained for neu (anti-c-Erb2/c–Neu, Calbiochem), Annexin V and PI according to the manufacturer’s protocol (BD Pharmingen). Flow cytometry was used to analyze the viability of neu positive cells [17, 30].

*IFN-γ ELISA*. Reprogrammed immune cells were cultured in complete medium with irradiated (140 Gy) tumor cells, ADR-treated dormant MMC or ADR+CQ-treated dormant MMC at a 10:1 ratio for 20 hrs. Supernatants were then collected and stored at −80°C until assayed. IFN-γ was detected using a Mouse IFN-γ ELISA kit (BD Biosciences), according to the manufacturer’s protocol [30].

### Statistical analysis

Data are summarized as means and standard errors of the mean (SEM) with differences between groups being illustrated with graphical data presented as mean ± SEM. Statistical comparisons were made using a one-tailed or two-tailed Student *t* test and a *p*-value < 0.05 was considered significant (^*^: < 0.05, ^**^: < 0.005. ^***^: < 0.0005, ^****^: < 0.00005).

## SUPPLEMENTARY MATERIALS FIGURES



## Abbreviations

ADR: Adriamycin
ATG5: Autophagy-related gene 5
BRCA1: Breast cancer gene 1
CQ: chloroquine
HSP70: Heat shock protein 70
IHC: Immunohistochemoistry
LAMP2A: Lysosomal membrane associated protein 2 A
MMC: neu-overexpressing mouse mammary carcinoma
TNBC: Triple negative breast cancer
