# Supplementary material for: Autophagy-deficient breast cancer shows early tumor recurrence and escape from dormancy
Source: Oncotarget. 2018 Apr 24;9(31):22113–22. doi: 10.18632/oncotarget.25197 (PMC5955162; doi:10.18632/oncotarget.25197)
Supplement: Supplementary file 1 [file oncotarget-09-22113-s001.pdf]

Autophagy-deficient breast cancer shows early tumor recurrence and escape from dormancy

SUPPLEMENTARY MATERIALS

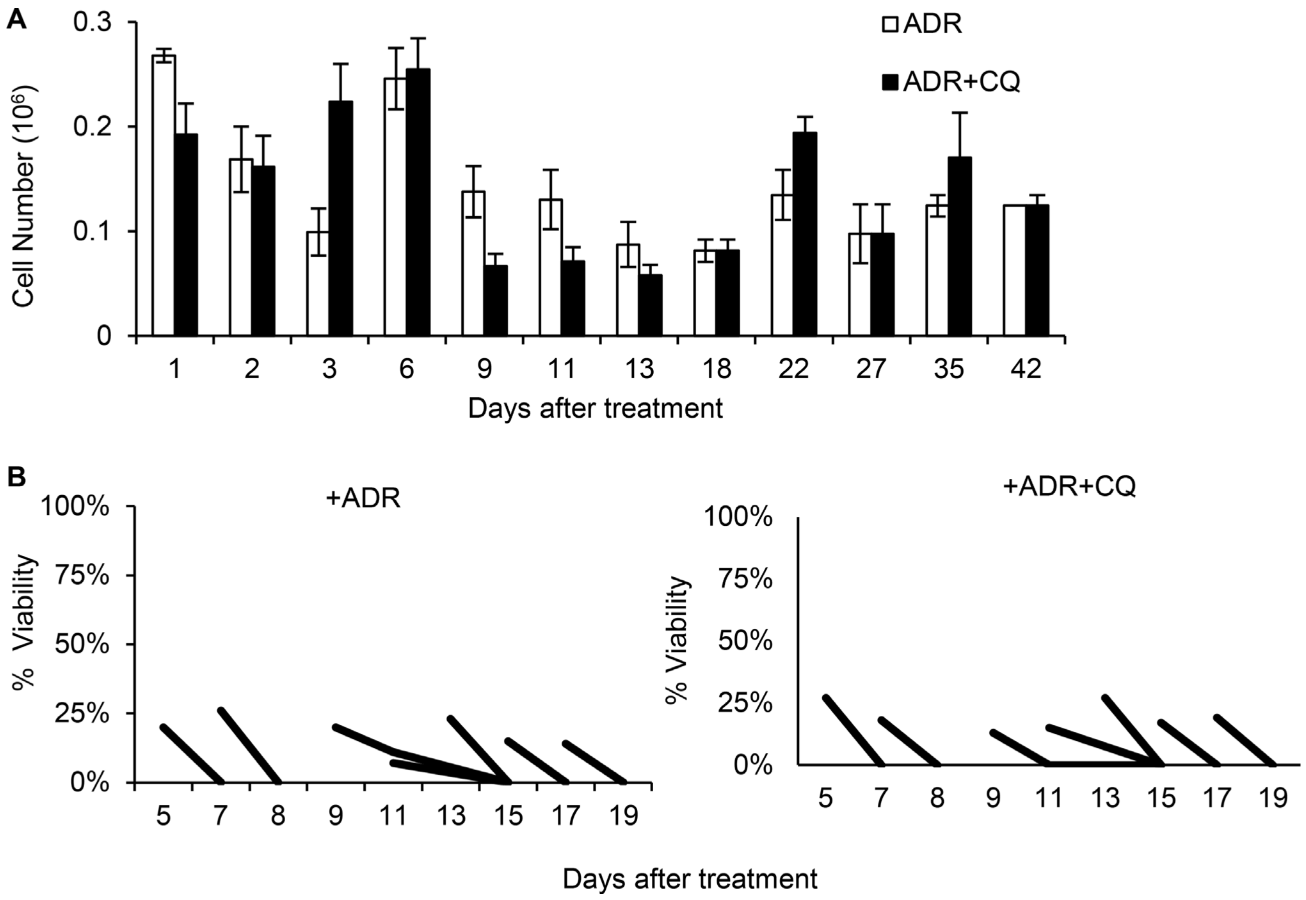

Supplementary Figure 1: ADR-induced dormant tumor cells produce floater apoptotic cells, *in vitro*. MMC tumor cells (3×10^6 cells/flask) were treated with 3 daily doses of ADR (1 uM for 2 hrs), with one group receiving CQ (10 uM) 3 hrs prior to and during ADR treatment. Both groups remained untreated for 3 weeks and 6 weeks, *in vitro*. (**A**) Floater cells were collected whenever culture medium was replaced and cell number and viability was assessed via trypan blue exclusion. Data represent 3 independent experiments and mean ± SEM. (**B**) Floater cells were cultured separately for 2–3 days each time they were collected, and assessed for viability 2–4 days later by using trypan blue staining.

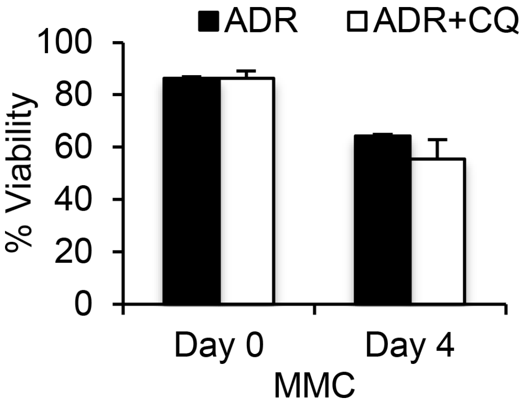

Supplementary Figure 2: A transient blockade of autophagy by CQ did not change the susceptibility of MMC to ADR treatment. MMC tumor cells were treated with ADR alone (1 uM ADR for 2 hrs) (ADR) or in the presence of CQ (10 uM 3 hrs before ADR and 2 hrs during ADR treatment) (ADR+CQ). Tumor cells were analyzed by Annexin v/PI staining prior to treatment (Day 0) or three days after the treatment (Day 4). Experiments were performed in triplicates.

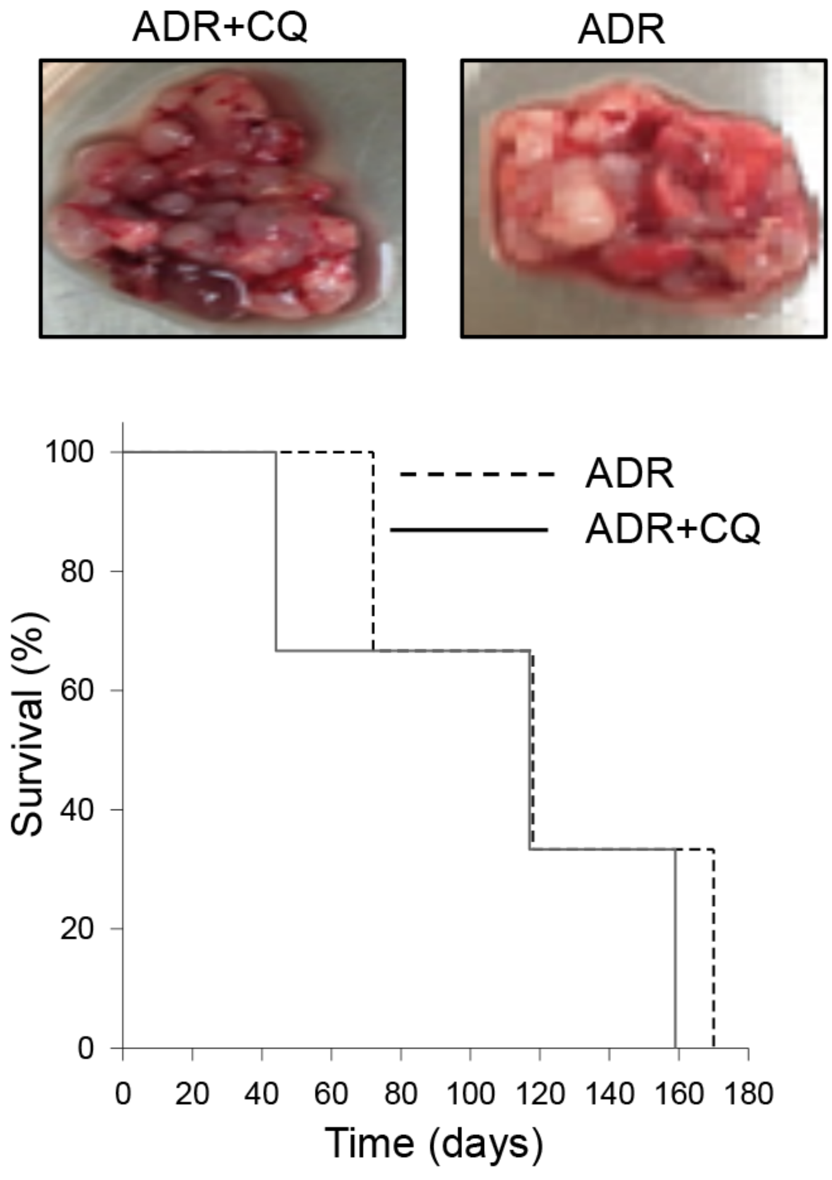

Supplementary Figure 3: A transient blockade of autophagy by CQ during ADR treatment fails to maintain tumor dormancy, *in vivo.* FVBN202 mice (*n* = 3/group) were challenged with MMC (i.v. injection of 1 million viable cells), and three days after tumor challenge animals were split into two groups: one group received 3 weekly treatments of ADR (i.v., 9 mg/kg), and another group received 3 weekly treatment of ADR + 60 mg/kg CQ (i.p.). Animals were sacrificed when they became moribund. Figure shows Kaplan-Meier survival curve and tumors in the lung.

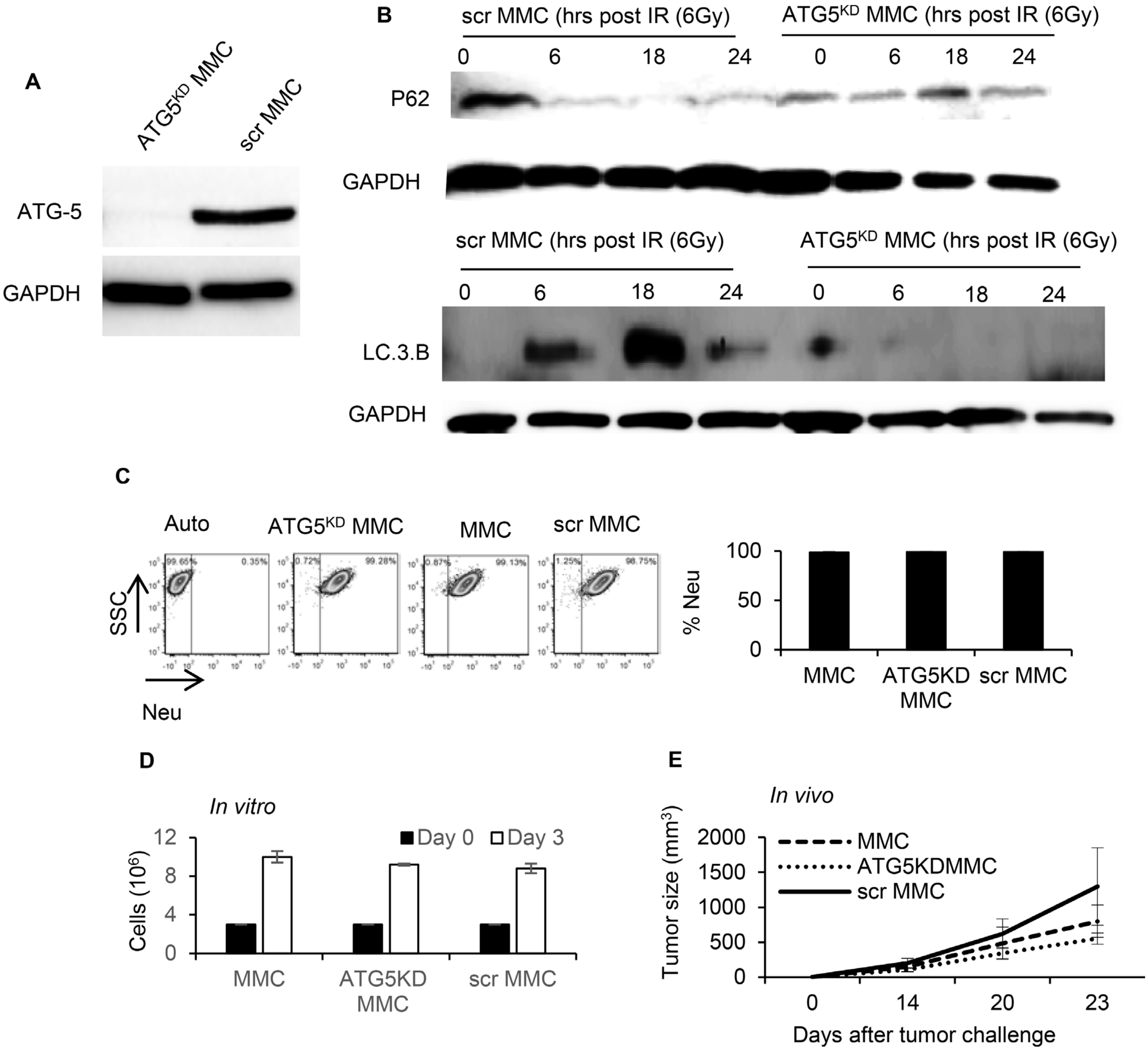

Supplementary Figure 4: ATG5 knockdown tumor cells and wild type MMC show a similar pattern of growth. MMC cells were stably transfected with lentivirus expressing shRNA against ATG5 to establish autophagy-deficient cells ($ATG5^{KD}$ MMC). Control MMC (MMC) were stably transfected with scrambled control vector as autophagy-competent cells (scr MMC). (**A**) Cell lysates were collected and used for immunoblotting against ATG5. (**B**) $ATG5^{KD}$ MMC and scr MMC were treated with IR (6G) and cells lysates were collected at 6, 18, 24 hrs post treatment. Autophagy was determined by degradation of p62 and accumulation of LC.3.B (**C**) Expression of Neu protein was determined on autophagy-competent control MMC (MMC or scr MMC) and autophagy-deficient MMC ($ATG5^{KD}$ MMC) using FACS analyses. (**D**) Tumor cell proliferation was determined in a 3-day culture using trypan blue exclusion. (**E**) FVBN202 mice ($n = 3$) were inoculated with autophagy-competent MMC (MMC or scr MMC) or autophagy-deficient MMC ($ATG5^{KD}$ MMC) (3×10^6 cell/mouse, s.c. inoculation), and tumor growth was monitored by using a digital caliper. Data represents triplicate experiments.
